# Supplementary material for: Exploring the effect of lexical inferencing and dictionary consultation on undergraduate EFL students’ vocabulary acquisition
Source: PLoS One. 2020 Jul 30;15(7):e0236798. doi: 10.1371/journal.pone.0236798 (PMC7392316; doi:10.1371/journal.pone.0236798)
Supplement: S1 Appendix — (DOCX) [file pone.0236798.s001.docx]

**Appendices**

**Appendix A:** List of target and control words used in the pre- and delayed post-test sessions. Target and control words were presented to participants in one list of 48 words, such that no more than three consecutive words were target or control words, respectively.

| **target words** | **control words** |
| --- | --- |
| 1.    remarkably | 1.    existence |
| 2.    to encounter | 2.    industrial |
| 3.    rapidly | 3.    patchy |
| 4.    defined | 4.    argumentative |
| 5.    astonished | 5.    consumption |
| 6.    neatly | 6.    to differentiate |
| 7.    to compete | 7.    an expedition |
| 8.    glamorous | 8.    to be stunned by |
| 9.    diverse | 9.    incompatible |
| 10.  to distribute | 10.  to incur |
| 11.  to appreciate | 11.  concept |
| 12.  interior | 12.  to vie |
| 13.  prejudices | 13.  to complain |
| 14.  intentional | 14.  arrogant |
| 15.  to tolerate | 15.  lurking |
| 16.  stimulation | 16.  intervention |
| 17.  intellectual | 17.  vendor |
| 18.  ethnicity | 18.  to urge |
| 19.  to observe | 19.  to contend |
| 20.  to be involved in | 20.  spontaneous |
| 21.  to combine | 21.  leash |
| 22.  perception | 22.  to exhibit |
| 23.  representation | 23.  to be timid |
| 24.  to influence | 24.  a tip |

**Appendix B:** Texts used in the two training sessions with target words underlined.

**Text 1:** Human societies - from the iceman to us

Imagine you were born some 300 years ago, in the year 1700. Although this is very recent in terms of the billions of years of the existence of planet Earth, you would still have been living in a remarkably different world. You would never have been to a shopping mall. You would never have encountered the world of cars, railways, airplanes, telephones, cameras, computers, and TVs. Welcome to the modern world!

Life has certainly changed in 300 years, and sociology was born out of a concern with this rapidly changing character of the modern, industrial world: with where we have come from and where we are heading. For sociologists, the term society means “all the people who interact in a defined space and shared culture”. In this sense, both a continent like Europe and specific individual countries such as Norway or Japan may be seen as society.

Even humans living thousands of years ago were members of early human societies. Evidence of this comes from the discovery of the Iceman. Examining the Iceman’s clothes, scientists were astonished at how advanced this ‘caveman’s’ society was. The iceman’s hair was neatly cut. He wore a skilfully sewn leather coat with a grass cape that provided even greater protection from the weather. It is estimated that he died some 5,300 years ago, before a great empire existed in Egypt and before any society in Europe built a single city.

**Text 2:** Marketing’s impact on consumers

For better or worse, we live in a world that is significantly influenced by marketers. We are surrounded by marketing stimuli in the form of advertisements, shops, and products competing for our attention and our cash. Much of what we learn about the world is controlled by marketers, whether through conspicuous consumption shown in glamorous magazine advertising or via the roles played by family members in TV commercials. Ads show us how we ought to act with regard to many diverse issues, including recycling, what we eat and drink, and even the types of house or car we desire.

In many ways, we are ‘at the mercy’ of marketers since we rely on them to sell us products that are safe and that perform as promised, to tell us the truth about what they are selling, and to price and distribute these products fairly. The role marketing plays in the creation and communication of popular culture is hard to ignore. However, many people fail to appreciate how much their view of the world – their film and music icons, the latest fashions in clothing, food, and interior design, and even the physical features that they find attractive in another person – is influenced by the marketing system. Product placement, whereby products and brands are used in popular movies or TV programs is an example of how companies command our attention.

**Text 3:** Family

The family is the most important agent of socialization because it represents the center of children’s lives. Babies are almost totally dependent on others, and the responsibility of meeting their needs almost always falls on parents and other family members. At least until the start of schooling, the family is responsible for teaching children cultural values, attitudes, and prejudices about themselves and others.

Family-based socialization is not entirely intentional. Children learn continuously from the kind of environment that adults create for them. Whether children learn to think of themselves as strong or weak, smart or stupid, loved or simply tolerated, and whether they believe the world to be safe or dangerous, largely stems from this early environment that adults create.

Parenting styles aside, parenting attention is important in the social development of children. Physical contact, verbal stimulation, and openness from parents and all others all encourage intellectual growth.

The family also confers on children a specific social position; that is, parents not only bring children into the physical world, they also place them in society in terms of race, ethnicity, religion, and class. In time, all of these elements become part of a child’s self-concept, or idea of him- or herself. Of course, some aspects of social position may change later on, but social standing at birth affects us throughout our lives.

**Text 4:** Principles that define the cognitive level of analysis

When people are thinking about how best to solve a mathematical problem, trying to remember the title of a book, observing a beautiful sunset, telling a joke or story that they have heard, or thinking about what to do tomorrow, they are involved in cognitive processing. Cognitive psychology is a branch of psychology which is concerned with the structure and function of the mind. Cognitive psychologists are involved in finding out how the human mind comes to know things about the world and how it uses this knowledge. Cognitive neuroscience combines knowledge about the brain with knowledge about cognitive processes.

The mind can be seen as a set of mental processes that are carried out by the brain. Cognitive processes include perception, thinking, problem-solving, memory, language and attention. The concept of cognition refers to such processes. Cognition is based on a person’s mental representations of the world, such as images, words, and concepts. These mental representations are based on experiences, for example, things that we can see, hear, feel or smell. People have different experiences and therefore they have different mental representation – for example, of what is right or wrong, or about what boys and girls can or cannot do. This will influence the way they think about the world and how they act in the world.
