# Supplementary material for: Exploring the effect of lexical inferencing and dictionary consultation on undergraduate EFL students’ vocabulary acquisition
Source: PLoS One. 2020 Jul 30;15(7):e0236798. doi: 10.1371/journal.pone.0236798 (PMC7392316; doi:10.1371/journal.pone.0236798)
Supplement: Attachment — Submitted filename: Review PONE D-19-34526.pdf [file pone.0236798.s002.pdf]

Review of the PLOS ONE Manuscript D-19-34526 – Exploring the effect of lexical inferencing and lexical translation on undergraduate EFL students' vocabulary acquisition

This study follows a within-participants experimental design to analyse and compare the effectiveness of two vocabulary learning strategies, i.e., lexical inferencing and dictionary consultation, in the vocabulary development of undergraduate EFL learners. The participants had to learn a total of 24 target words using these two vocabulary strategies (12 words in each) over two training sessions. After the treatment, participants had to complete a translation task including the 24 target words and 24 control words, a vocabulary size checklist and a vocabulary strategies questionnaire. Generalised linear models showed that participants' knowledge of the target words improved to a similar extent under both learning conditions, suggesting that the two strategies have a comparable learning effect on vocabulary development. This learning was also affected by the participants' vocabulary size, with larger breadth of vocabulary leading to larger learning gains in both conditions. The authors also claim that learners' vocabulary size increased as a result of the treatment and discuss the factors that might have led to this increase.

This study is interesting and generally well though through, and there is certainly merit in examining the effect of these two very popular learning strategies in L2 vocabulary development. However, the manuscript in its current form does not meet the quality requirements to warrant publication in PLOS ONE. The paper attempts to address many research questions, which consequently has affected the coherence of the paper and the discussion of the main aim. More importantly, there are certain methodological issues that required more rigorous control and consideration and have affected the quality of the results and the discussion.

These and other issues are described below in more detail, along with comments and suggestions on how they might be addressed in order to warrant publication in future attempts.

Literature Review

One of the first matters that arose when reading this manuscript was the terminology employed to refer to the two learning strategies. While 'lexical inferencing' is sometimes used in the field interchangeably with 'guessing from context', I believe that using the term 'lexical translation' to refer to 'dictionary consultation' is misleading and indeed incorrect. Lexical translation involves students encountering the L1 form of a word and producing its L2 form, or vice-versa. In consulting a dictionary, learners do not have to recall any knowledge, as it is already provided to them, but simple search for the correct answer. Thus, for the sake of accuracy, the term 'lexical translation' should be avoided throughout the paper and instead refer to 'dictionary use' in future publication attempts.

The literature review in general requires some reorganisation and refocus in order to lead the reader more coherently to the main research question of the study. For example, since the key objective of the paper is comparing two vocabulary strategies, the taxonomy mentioned in line 46 should be discussed more thoroughly, making comparisons between other learning strategies and the two adopted in this study, and then concluding why these two and no other strategies were selected in this research.

Regarding dictionary use, the literature review should discuss the differences between using monolingual or bilingual dictionaries as vocabulary learning strategies according to research. This is a key strategy in the study, and thus should be thoroughly reviewed, while focusing on the discussion of bilingual dictionaries, as it is the one used in this study. Also, for the studies reported in section starting in line 96, it would be useful for the reader if you could specify whether they were using mono or bilingual dictionaries.

Section starting in line 135 does not link very well to the main aim of the essay, and thus I believe that it could be removed and this space dedicated to elaborate more on the key sections.

Section starting on line 148 is very interesting and relevant to the VLS topic. However, the location breaks down the flow of ideas and diverts attention from the key research question in the study. Thus, I believe that it would lead to a more focused and coherent literature review section if this idea was discussed in the VLS section at the beginning of the literature review, and then specifying the different processing depths for each of the two target strategies in the sections under their terms (sections 2 and 3). More importantly, since this is a lexical study, the discussion of processing depth must review the two key theories in the field regarding lexical engagement and processing: the Involvement Load Hypothesis (Hulstijn and Laufer

2001) and the Technique Feature Analysis (Nation and Webb 2011). Then the two target strategies should be analysed based on these two theories and the results of the study discussed in light of their different types and levels of engagement.

Finally, the references used in the study seem rather outdated (Kroll et al 1988; Carton, 1971). It is good practice to cite seminal papers, but I believe that more updated research in vocabulary learning strategies would improve the quality of the literature review and the discussion sections.

Methodology

The general design of the study is adequate and well thought through, and the analyses performed are appropriate to answer the research questions. Yet, one of the issues that have affected the quality of the study is its general focus. It attempts to cover too many research questions, some of which are not theoretically or practically justified. In particular, RQs 4 and 5 seemed to me slightly off track. I think the potential future study attempt would be significantly improved if it concentrated on the first 3 RQs.

Another important methodological matter concerns the instruments and materials selected. First of all, the vocabulary size test chosen for this study is the XK_Lex. While I understand why this test might have been appropriate in that it is culturally targeted to your participants, checklists do not require learners to demonstrate knowledge. This means that the results are subject to a great extent to the participants' understanding of "knowing" a word. Thus, the estimate of size provided is less reliable than if you had used a more standardised and reliable test, such as the VLT (Schmitt et al., 2001) or VST (Nation and Berglar, 2007).

You have carefully controlled for the 48 target words in this study (24 for treatment and 24 for control) in terms of frequency, word length, PoS and derivational complexity. However, the list of target words should be included in a table or appendix for the reader to better interpret their characteristics and the results.

Regarding the texts, very little information is provided about them, despite their immense importance and effect in successful lexical inferencing. In the literature review you mention the need for large lexical coverage to be able to understand a text and infer unknown words successfully (e.g., Nation 2006). However, there is no mention in the methodology about how the authors ensured that the texts selected for the treatment met these lexical coverage

requirements in relation to the participants' vocabulary knowledge. If this was something the authors controlled for, it should be reported in detail giving its potential effect on the lexical inferencing condition. If not controlled for, the study should be redesigned and the data collected again ensuring appropriate lexical coverage in the target texts based on the participants' vocabulary size. Similarly, in line 69 the authors start discussing the types of cues that affect lexical inferencing. Yet, it was not reported how these cues were controlled for around the target words and the control words. The 4 texts employed should be provided as appendices for the reader to evaluate and to understand which target words appeared in each text and which lexical and contextual cues each had. Moreover, the dictionary employed by learners to complete the treatment task should be cited.

As for the word translation task, the authors do not explain what kind of translation it required. It is assumed that it was L2 to L1, but this should have been explicitly mentioned. Moreover, According to vocabulary research, the different direction that a translation task can take (L2 → L1 or L1 → L2) affect the difficulty it poses for learners. In particular, the translation direction employed in this study (L2 form given and requesting L1 meaning to be provided) is considered easier for learners (e.g. Laufer and Goldstein, 2004), what can have influenced the results. Thus, in future publication attempts, the findings should be discussed in light of this idea.

Regarding the design, the study does not include a delayed post-test session at least one week after the treatment. This limits the extent to which the proposed vocabulary gains in this study can be considered durable. I would recommend that the authors add a delayed post-test in future attempts to re-conduct this study, which could inform about the actual longer-term gains from these short sessions as well as potential differences between the two learning strategies regarding retention (i.e., the immediate gains from both can be similar, but maybe the deeper engagement required for lexical inferencing leads to more retention?).

Finally, there is no mention of the time given and taken by the participants to complete the inferencing and dictionary use tasks. This information is needed for the reader to evaluate the significance and extent of the learning gains.

Results and discussion

The above methodological issues have inevitably affected the results of the study and the discussion.

Firstly, while the participants have been reported as advanced learners by the authors and the learners' self-assessed proficiency, the results of the vocabulary size test shows that they only know 3369 lemmas. Based on this, the authors might want to reconsider reporting them as advanced learners.

More importantly, this finding raises concerns regarding the difficulty of the four texts employed in the study and the level of lexical coverage of these texts by the participants. Did the texts meet the 98% lexical coverage required for successful guessing from context? Since the texts have not been provided, it has not been possible for me as a reviewer to check this, but it is likely that the lexical inferencing findings might have been affected by the participants' low vocabulary level. This should be checked by the authors and discussed in future publication attempts.

The results of learning gains from lexical inferencing and dictionary use are interesting, and their discussion would benefit from relating the findings to the Involvement Load Hypothesis, as suggested above. Moreover, I think it would enhance the discussion if the authors reported which words were typically learnt by most participants and interpret the findings in light of the contextual and linguistic cues included in the relevant texts were they were learnt. Did those words share common cues in the text? If so, which?

Regarding the discussion of the findings from RQ2, authors state "Our findings indicate that both VLS methods lead to higher learning than mere incidental learning". I think it is misleading to use the term 'incidental learning' here when referring to control words, since this study was not comparing deliberate vs. incidental learning of words. In incidental learning situations there are not any enhanced/underlined words in the texts encountered by learners, whereas in your texts the target words were underlined. Thus, even though the control words were not enhanced in any manner, this cannot be considered lack of "incidental learning" as the students' attention was drawn to some specific words, and thus they probably did not even notice non-underlined words. Therefore, comparing the results of vocabulary learning strategies to incidental learning in this study is not accurate.

Finally, the results regarding the increase of vocabulary size and factors influencing this seem rather problematic to me. It seems quite unlikely that learners' vocabulary size increases about 500 lemmas in 5 weeks, particularly given that the participants average vocabulary size is 3331 lemmas after multiple years of English learning and University instruction in English. A more likely explanation for this result would be the test-retests effect and the fact that the test

involves only a checklist and no demonstration of knowledge, which means that participants might tick extra words in the post-test because they remember seeing them in the pre-test session even if they are unsure of their meaning or use. Thus, I believe that in future publication attempts the findings from RQs 4 and 5 should be taken with caution, and the discussion in lines 651 to 670 revised.

Overall, while the main research questions and ideas covered in this study are interesting and worth investigating, the current manuscript requires significant methodological changes for it to be considered for publication in future attempts.

Minor considerations and typos.

Abstract → the abstract says that 63 advanced learners participated in the study, but line 194 states that the final pool included only 61. The abstract information should include the final number of analysed data.

Line 48 → add "employed ***to*** remember"

Line 60 → add "***lexical*** text coverage"

Lines 66, 69 and throughout the text → number of reference in brackets does not match those in the bibliography.

Line 85 → add "***bilingual*** dictionary usage"

Line 208 → add "current ***study***"

Line 211 → since you state later on (line 341) that the results of these VLS questionnaire are beyond the scope of this study I wonder whether you should mention it here at all. It can cause confusion to the reader and doesn't add anything to this study.

Line 257 → insure → ***ensure***

Line 428 → knew during training → ***before*** training

Line 476 and 478 → ***vocabulary size***, not 'overall vocabulary knowledge'

Lines 728-733 → there is repetition of the same study (Tanyer and Ozturk)
