# Supplementary material for: Exploring the effect of lexical inferencing and dictionary consultation on undergraduate EFL students’ vocabulary acquisition
Source: PLoS One. 2020 Jul 30;15(7):e0236798. doi: 10.1371/journal.pone.0236798 (PMC7392316; doi:10.1371/journal.pone.0236798)
Supplement: Attachment — Submitted filename: Reviewers comments - R1.pdf [file pone.0236798.s004.pdf]

First of all, I would like to commend the authors for the substantial work put towards the review and rewriting of this manuscript. The authors have taken into account, and successfully addressed, most of the comments and recommendations by both reviewers, and this has certainly improved the manuscript. In particular, having accepted the recommendation to focus on the first 3 RQs has provided the paper with a clearer focus and eliminated some issues that the study's methodological decisions posited for answering the previous RQs 4 and 5 (i.e., claims about vocabulary size improvements based on a checklist test).

The new explanation provided by the authors on the selection of the two VLSs examined in the study is clearer for the reader and convincing.

Moreover, the added analysis testing the relationship between lexical coverage (i.e., vocabulary size) and guessing from context adds considerable value to the results and discussion of the findings. However, beware of my comments on this matter below, which show that this cannot be consider an exploration of the relationship between *lexical coverage* and guessing, but rather between *vocabulary size* and guessing (i.e., lexical coverage refers to the percentage of running words known by the learner in a text, not to a specific vocabulary size, as this would change depending on the authenticity of the text). See my comments below for further details.

The authors have also taken into consideration the reviewer's suggestion to include the ILH and TFA theories in the literature review to explain the processing depth and elaboration required by the two VLSs. These lexical theories are relevant to the aim of the study and have provided a useful insight for the description of the two VLSs and as well as for the discussion of results. In lines 131-149 and 185-201, the authors have evaluated the lexical inferencing and dictionary consultation strategies (respectively) in light of the ILH and TFA. This provides cohesion to the manuscript, as well as an appropriate framework for the comparison of the two strategies in terms of cognitive processing. Yet, I believe that these theories could be exploited somewhat more in the discussion section. In particular, it would be interesting to include a remark suggesting that the similar ILH and TFA processing value reported by the two VLSs (e.g., both obtaining a score of 4 in light of the ILH) might be a possible explanation for the finding that both strategies led to similar levels of word learning and retention.

While most of the comments have been appropriately addressed and do not require further significant changes, there are some that still need some consideration before the manuscript can be ready for publication.

Firstly, the authors have provided valid reasons (mainly practical) for the selection of the XK_Lex test instead of other more established tests, such as the VLT and VST, which are the reasons also made by previous research employing this test. However, although the authors disagree with the reviewer's claim that this test does not provide demonstration of knowledge, I feel that this is still the case for the test, and also a main reason why most vocabulary studies do not use checklists to measure a learners' vocabulary size. This test requires students to tick whether they know a word or not, and indeed includes non-words and applies a formula to account for potential random ticking of unknown words. Nevertheless, this test involves simply *self-report* of knowledge, not actual *demonstration* of knowledge, which means that learners' personal characteristics affect the results in this test even more than in other tests that do require some type of demonstration of knowledge (e.g. VST, VLT). This might actually have also been the case in the current study, given the finding that the vocabulary size of the students, all enrolled in English major degrees, varied greatly with some learners reporting knowing 1200 words and others 7600 words. This could be reflecting the different personalities of learners, with some ticking a word only when they were absolutely sure that they knew the form and the meaning of the words, and others ticking words that simply looked familiar to them. Thus, these very different size results could have potentially been an artifact of having employed this self-report checklist test instead of another measure that requires demonstrating knowledge of the form or meaning of the word by means of recall or recognition.

As the authors report, though, all tests have their own limitations, and the characteristics of the XK_Lex might have made it adequate for the current study (i.e., targeted to the specific learner population, uses lemma as word counting unit, and is administered in less than 10 minutes). Nevertheless, I think that it is important that the authors address as a limitation of the study the main weakness of this test, which is that it only involves self-report of knowledge and thus answers can represent not only different vocabulary sizes but also personality traits.

Secondly, the analysis and discussion of the relationship between lexical coverage and inferencing require some modifications. As stated above, this is really an analysis of the relationship between vocabulary size and inferencing that can inform lexical coverage research. The authors make the following claim in the discussion: "our results do not support the claim that 98% of text coverage requiring a vocabulary size of 8000 to 9000 words is needed for successful inferencing [31,32].". However, this claim is incorrect. Nation (2006) argues that 8,000-9,000 word families are needed for 98% lexical coverage and *successful understanding* of *authentic* texts on a wide variety of topics and disciplines. He also claims that 95% lexical

coverage is enough for adequate comprehension of the text (~5,000-6,000 word families for authentic texts on a variety of topics). It is, thus, expected by vocabulary researchers and practitioners that when a text is developed for learning purposes, and thus adapted in difficulty, 98% lexical coverage (i.e., knowing 98% of the words in the text) will be achieved with potentially much lower vocabulary sizes. For example, it is possible to create a whole text employing only words within the most frequent 1000 words in a language, and thus a vocabulary size of about 1000 word families would already provide this 98% lexical coverage.

The texts included in the current study are taken from English textbooks, and thus are *contrived* or *semi-authentic* texts (indeed, the authors themselves mention that some technical words in the texts were changed to adapt it to the learner's proficiency). Therefore, a vocabulary size of less than 8,000 word families would be enough to understand these texts, since they are designed and adapted for English learners of lower vocabulary sizes. This does not mean that the 98% lexical coverage for successful independent comprehension of the text is not needed. Rather, it means that the chosen texts in this study are easier than authentic texts, and thus 95-98% lexical coverage can be achieved with far less than 8,000 word families.

In order to demonstrate this, I conducted a brief lexical coverage analysis on the 4 texts employed in this study, specifically on their required vocabulary size for achieving 95-98% lexical coverage as suggested by Nation (2006). I employed the vocabulary profiler in the publicly-available software Lextutor.

Text 1: In this text, over 95% lexical coverage was achieved only with knowledge of 3,000 word families. No word in the text was beyond the 5,000 word family level (see figure below).

Freq. Level	Families (%)	Types (%)	Tokens (%)	Cumul. token (%)
K-1 :	94 (75.2)	107 (72.79)	217 (82.2)	82.2
K-2 :	21 (16.8)	23 (15.65)	28 (10.6)	92.8
K-3 :	7 (5.6)	7 (4.76)	7 (2.7)	95.5
Coverage 95				
K-4 :	2 (1.6)	3 (2.04)	3 (1.1)	96.6
K-5 :	1 (0.8)	1 (0.68)	1 (0.4)	97.0

Text 2: 4,000 word families were enough for 98% lexical coverage, and no word in the text was beyond the 5,000 word families (see figure below).

Freq. Level	Families (%)	Types (%)	Tokens (%)	Cumul. token (%)
K-1 :	77 (63.6)	89 (65.44)	196 (79.0)	79.0
K-2 :	27 (22.3)	29 (21.32)	35 (14.1)	93.1
K-3 :	10 (8.3)	10 (7.35)	10 (4.0)	97.1
		Coverage 95		
K-4 :	4 (3.3)	4 (2.94)	4 (1.6)	98.7
		Coverage 98		
K-5 :	3 (2.5)	3 (2.21)	3 (1.2)	99.9

Text 3: 4,000 word families were enough for 98% lexical coverage (actually, 98% coverage was almost achieved by the first 3,000 word families), and no word in the text was beyond the 5,000 word families (see figure below).

Freq. Level	Families (%)	Types (%)	Tokens (%)	Cumul. token (%)
K-1 :	89 (71.2)	100 (72.99)	199 (81.9)	81.9
K-2 :	23 (18.4)	23 (16.79)	30 (12.3)	94.2
K-3 :	9 (7.2)	9 (6.57)	9 (3.7)	97.9
		Coverage 95		
K-4 :	3 (2.4)	3 (2.19)	3 (1.2)	99.1
		Coverage 98		
K-5 :	1 (0.8)	1 (0.73)	2 (0.8)	99.9

Text 4: 98% lexical coverage was achieved with 4,000 word families, and only two words were beyond this level (*cognition* and *neuroscience*) (see figure below).

Freq. Level	Families (%)	Types (%)	Tokens (%)	Cumul. token (%)
K-1 :	70 (69.3)	77 (66.96)	188 (77.0)	77.0
K-2 :	19 (18.8)	21 (18.26)	32 (13.1)	90.1
K-3 :	9 (8.9)	12 (10.43)	13 (5.3)	95.4
		Coverage 95		
K-4 :	1 (1.0)	1 (0.87)	7 (2.9)	98.3
		Coverage 98		
K-5 :				
K-6 :				
K-7 :				
K-8 :	1 (1.0)	1 (0.87)	2 (0.8)	99.1
K-9 :				
K-10 :				
K-11 :				
K-12 :				
K-13 :	1 (1.0)	1 (0.87)	1 (0.4)	99.5

Thus, the above claim made by the authors should be reviewed, since your study cannot inform about whether 8,000-9,000 word families are needed to understand the majority of an authentic text. Rather, your study shows that, for these texts, learners required only a vocabulary size of between 3-4,000 word families in order to achieve the recommended lexical coverage for successful text comprehension (i.e., 98%). Thus, the results of this study seem to be in line with this claim that 95-98% lexical coverage of a text (whatever vocabulary size that represents in the contrived texts) is needed for adequate comprehension and inferencing to occur.

Finally, regarding the results, the authors rightly compare the known words in the pre-test and the delayed post-test, and subtract the former from the latter to report relative gains. However, there is no report of the words known and unknown by the participants during the training sessions. For the sake of transparency, it would be interesting to report how many of the target words the learners reported as “known” during the training session, and how this compares with the learning retained 2 weeks later. This would allow the readers to better understand whether the learners retained the same amount of knowledge they reported during the training or indeed this knowledge was significantly higher in the delayed post-test, which would emphasise the effectiveness of the VLSs.

Overall, the authors have made significant changes and improvements on the reviewed manuscript. Thus, once the comments made above are addressed, the paper should be ready for publication.

line 110: *8000 and 9000 words* --> word families. Also, it should be made explicit in the manuscript that these numbers were proposed for adequate comprehension of authentic texts (i.e., designed for the consumption of speakers of that language, not for learners) on a variety of topics. Thus, the vocabulary size to achieve 98% coverage in semi-authentic or contrived texts is expected to be much lower. This is important to be included also in the relevant discussion section.
