# Supplementary material for: Exploring the effect of lexical inferencing and dictionary consultation on undergraduate EFL students’ vocabulary acquisition
Source: PLoS One. 2020 Jul 30;15(7):e0236798. doi: 10.1371/journal.pone.0236798 (PMC7392316; doi:10.1371/journal.pone.0236798)
Supplement: Attachment — Submitted filename: Reviewers comments - R1_v3.pdf [file pone.0236798.s005.pdf]

We would like to thank the reviewers again for their careful reading of our manuscript and their additional feedback. We believe that the feedback has again substantially improved the paper, we hope that we have addressed all remaining concerns and that the paper is now ready for publication.

Reviewer #1: The authors have comprehensively addressed both reviewers' comments and have made significant revisions that have improved the manuscript considerably. I am happy to recommend that this paper be accepted. There are just a handful of minor textual clarifications that I believe need addressing to finalise the paper for publication.

Thank you very much. We are glad to hear that we have addressed most of the comments and recommendations.

1) I think a little reworking of the text is needed from lines 107 and 720. In these sections, the authors discuss the estimated lexical knowledge required for successful inferencing, citing Nation, who suggests 98% coverage needed for lexical inferencing, and Laufer & Ravenhorst-Kalovski, who say knowledge of 8-9000 words needed to achieve this coverage. Were these researchers were talking about a specific type of text, that is, academic texts? In other words, to read typical academic texts, knowledge of 8-9000 words is required to reach 98% coverage. The authors do not mention the genre but should do so in the section starting line 107.

Thank you for highlighting this issue. We have now addressed this in the lexical inferencing section, where we now mention that Nation (2006) based the 98% lexical coverage estimate for adequate comprehension on a variety of written and spoken texts, such as novels and newspaper articles, while Laufer & Ravenhorst-Kalovski (2010) based their estimates on academic texts. We have also added information from Nation (2006) suggesting a lower threshold of about 3000 word families for simplified texts, as our texts fall into that category. In light of this lower threshold for simplified texts and reviewer 2's comments, we have also amended our discussion on text coverage.

Also, in the discussion from line 720 the authors suggest that 98% coverage is perhaps not required for successful inferencing because the participants were successful even though they had average vocabulary sizes of 3-4000 words, not 8-9000 words. But then from line 730 (and also from 429 where the texts are described in the methodology) the authors suggest that the texts used in this study were most likely simpler than typical academic texts, which is why participants could achieve successful inferencing. It is unclear to me whether the 98% coverage at 8-9000 words really applies in this case. Basically 98% coverage depends specifically on the lexis present in the texts and the learners' lexical knowledge (a beginner learner can reach 98% coverage of some children's books). Without reporting the specific number of words and their frequencies in the texts, it is difficult to convincingly challenge the claims by previous researchers.
The argumentation in these sections should therefore be clarified.

Thank you for noting this issue. 98% text coverage at 8-9000 word families does indeed not apply in this case. As reviewer 2 kindly provided information about text coverage for our texts through the vocabulary profiler in Lextutor, we can now estimate the number of word families needed for 98% text coverage for the four texts that we chose. Specifically, Lextutor suggests that 4000 word families are needed for 98% text coverage for our texts. We now mention this information in the methods section, and we have changed the discussion on text coverage accordingly. Specifically, considering that knowledge of fewer word families is needed for 98% text coverage for out texts compared to authentic materials, we have now weakened our claims in the discussion section and hope that our amended argument, which is now based on information specific to our texts, is more convincing.

2) I found the following text (from line 690) somewhat misleading as well: "the current results are fully compatible with the idea that just guessing can lead to incorrect learning as we found that participant [-s missing here] who were better at guessing the correct meanings of target words during the training also showed higher learning than participants who were less successful at guessing correctly during the training."
It seems to me that the evidence does not suggest that 'guessing can lead to incorrect learning' but instead suggests that 'guessing can lead to no learning'. The authors would may wish to modify the text to resolve this issue.

Thanks so much for pointing this out. We have now weakened the claim by rephrasing the sentence so that we now suggest that "guessing can lead to no learning and possibly even incorrect learning". In addition, we have added the following sentence to explain how our results are compatible with incorrect learning: "The above result means that, on the flip-side, participants who more frequently guessed incorrectly during training showed less learning, and the reason for this may be that the incorrect guesses during training lead them to learn an incorrect meaning for some items." We hope that this clarifies the issue.

3) From line 695: "strategies should explicitly be taught or should arise naturally in the language learner" – I think the latter part needs changing to "…or should be adopted naturally' or similar. It seems odd to say strategies arise in someone.

Thanks so much for pointing this out. We have replaced "should arise" with "should be adopted".

Reviewer #2:

I would like to commend the authors for their huge efforts to review and rewrite this manuscript. I feel that these have improved the paper considerably.

First of all, I would like to commend the authors for the substantial work put towards the review and rewriting of this manuscript. The authors have taken into account, and successfully addressed, most of the comments and recommendations by both reviewers, and this has certainly improved the manuscript. In particular, having accepted the recommendation to focus on the first 3 RQs has provided the paper with a clearer focus and eliminated some issues that the study's methodological decisions posited for answering the previous RQs 4 and 5 (i.e., claims about vocabulary size improvements based on a checklist test).

Thank you very much. We are glad to hear that we have addressed most of the comments and recommendations.

The new explanation provided by the authors on the selection of the two VLSs examined in the study is clearer for the reader and convincing.

Moreover, the added analysis testing the relationship between lexical coverage (i.e., vocabulary size) and guessing from context adds considerable value to the results and discussion of the findings. However, beware of my comments on this matter below, which show that this cannot be consider an exploration of the relationship between *lexical coverage* and guessing, but rather between *vocabulary size* and guessing (i.e., lexical coverage refers to the percentage of running words known by the learner in a text, not to a specific vocabulary size, as this would change depending on the authenticity of the text). See my comments below for further details.

Thank you for pointing this out. Our assumption was that for any given text, a larger vocabulary size would correspond to greater lexical coverage. We do, however, realize that the relationship between text coverage and vocabulary size is not that straightforward, and – as the reviewer rightly points out – depends on the level of the text. We have therefore deleted the reference to text coverage when describing and reporting this particular additional analysis.

The authors have also taken into consideration the reviewer's suggestion to include the ILH and TFA theories in the literature review to explain the processing depth and elaboration required by the two VLSs. These lexical theories are relevant to the aim of the study and have provided a useful insight for the description of the two VLSs and as well as for the discussion of results. In lines 131-149 and 185-201, the authors have evaluated the lexical inferencing and dictionary consultation strategies (respectively) in light of the ILH and TFA. This provides cohesion to the manuscript, as well as an appropriate framework for the comparison of the two strategies in terms of cognitive processing. Yet, I believe that these theories could be exploited somewhat more in the discussion section. In particular, it would be interesting to include a remark suggesting that the similar ILH and TFA processing value reported by the two VLSs (e.g., both obtaining a score of 4 in light of the ILH) might be a possible explanation for the finding that both strategies led to similar levels of word learning and retention.

Thanks so much for the suggestion. We have added the following remark as requested: "In fact, the similar levels of processing depth of both VLS according to the Involvement Load Hypothesis and Technique Feature Analysis might be a possible explanation for the finding that both strategies led to similar levels of word learning and retention."

While most of the comments have been appropriately addressed and do not require further significant changes, there are some that still need some consideration before the manuscript can be ready for publication.

Firstly, the authors have provided valid reasons (mainly practical) for the selection of the XK_Lex test instead of other more established tests, such as the VLT and VST, which are the reasons also made by previous research employing this test. However, although the authors disagree with the reviewer's claim that this test does not provide demonstration of knowledge, I feel that this is still the case for the test, and also a main reason why most vocabulary studies do not use checklists to measure a learners' vocabulary size. This test requires students to tick whether they know a word or not, and indeed includes non-words and applies a formula to account for potential random ticking of unknown words. Nevertheless, this test involves simply *self-report* of knowledge, not actual *demonstration* of knowledge, which means that learners' personal characteristics affect the results in this test even more than in other tests that do require some type of demonstration of knowledge (e.g. VST, VLT). This might actually have also been the case in the current study, given the finding that the vocabulary size of the students, all enrolled in English major degrees, varied greatly with some learners reporting knowing 1200 words and others 7600 words. This could be reflecting the different personalities of learners, with some ticking a word only when they were absolutely sure that they knew the form and the meaning of the words, and others ticking words that simply looked familiar to them. Thus, these very different size results could have potentially been an artifact of having employed this self-report checklist test instead of another measure that requires demonstrating knowledge of the form or meaning of the word by means of recall or recognition.

As the authors report, though, all tests have their own limitations, and the characteristics of the XK_Lex might have made it adequate for the current study (i.e., targeted to the specific learner population, uses lemma as word counting unit, and is administered in less than 10

minutes). Nevertheless, I think that it is important that the authors address as a limitation of the study the main weakness of this test, which is that it only involves self-report of knowledge and thus answers can represent not only different vocabulary sizes but also personality traits.

Thank you for this comment. We have now added information to the methods section that mentions this limitation of the XK_Lex test. In addition, we now also discuss this potential limitation of the XK_Lex test in quite a bit of detail in the discussion section. We draw on the previous literature on self-reported vocabulary knowledge as well as on additional analyses from our training sessions that show that during training participants over-reported their target word knowledge to a small to moderate extent, but were highly consistent in their over-reporting, with self-reported and actual target word knowledge being highly correlated. We tentatively suggest that our particular group of participants may have also consistently slightly over-reported their vocabulary knowledge in the XK_Lex test. We hope that this additional discussion has resolved the issue to the reviewer's satisfaction. Please also note that we accidentally reported that the XK_Lex contains 80 real words. It actually contains 100 real words and we have now corrected this in the methods section.

Secondly, the analysis and discussion of the relationship between lexical coverage and inferencing require some modifications. As stated above, this is really an analysis of the relationship between vocabulary size and inferencing that can inform lexical coverage research. The authors make the following claim in the discussion: "our results do not support the claim that 98% of text coverage requiring a vocabulary size of 8000 to 9000 words is needed for successful inferencing [31,32].". However, this claim is incorrect. Nation (2006) argues that 8,000-9,000 word families are needed for 98% lexical coverage and *successful understanding* of *authentic* texts on a wide variety of topics and disciplines. He also claims that 95% lexical coverage is enough for adequate comprehension of the text (~5,000-6,000 word families for authentic texts on a variety of topics). It is, thus, expected by vocabulary researchers and practitioners that when a text is developed for learning purposes, and thus adapted in difficulty, 98% lexical coverage (i.e., knowing 98% of the words in the text) will be achieved with potentially much lower vocabulary sizes. For example, it is possible to create a whole text employing only words within the most frequent 1000 words in a language, and thus a vocabulary size of about 1000 word families would already provide this 98% lexical coverage.

The texts included in the current study are taken from English textbooks, and thus are *contrived* or *semi-authentic* texts (indeed, the authors themselves mention that some technical words in the texts were changed to adapt it to the learner's proficiency). Therefore, a vocabulary size of less than 8,000 word families would be enough to understand these texts, since they are designed and adapted for English learners of lower vocabulary sizes. This does not mean that the 98% lexical coverage for successful independent comprehension of the text is not needed. Rather, it means that the chosen texts in this study are easier than authentic texts, and thus 95-98% lexical coverage can be achieved with far less than 8,000 word families.

In order to demonstrate this, I conducted a brief lexical coverage analysis on the 4 texts employed in this study, specifically on their required vocabulary size for achieving 95-98% lexical coverage as suggested by Nation (2006). I employed the vocabulary profiler in the publicly-available software Lextutor.

Text 1: In this text, over 95% lexical coverage was achieved only with knowledge of 3,000 word families. No word in the text was beyond the 5,000 word family level (see figure below).

Freq. Level	Families (%)	Types (%)	Tokens (%)	Cumul. token (%)
K-1 :	77 (63.6)	89 (65.44)	196 (79.0)	79.0
K-2 :	27 (22.3)	29 (21.32)	35 (14.1)	93.1
K-3 :	10 (8.3)	10 (7.35)	10 (4.0)	97.1
		Coverage 95		
K-4 :	4 (3.3)	4 (2.94)	4 (1.6)	98.7
		Coverage 98		
K-5 :	3 (2.5)	3 (2.21)	3 (1.2)	99.9

Text 2: 4,000 word families were enough for 98% lexical coverage, and no word in the text was beyond the 5,000 word families (see figure below).

Freq. Level	Families (%)	Types (%)	Tokens (%)	Cumul. token (%)
K-1 :	94 (75.2)	107 (72.79)	217 (82.2)	82.2
K-2 :	21 (16.8)	23 (15.65)	28 (10.6)	92.8
K-3 :	7 (5.6)	7 (4.76)	7 (2.7)	95.5
		Coverage 95		
K-4 :	2 (1.6)	3 (2.04)	3 (1.1)	96.6
K-5 :	1 (0.8)	1 (0.68)	1 (0.4)	97.0

Text 3: 4,000 word families were enough for 98% lexical coverage (actually, 98% coverage was almost achieved by the first 3,000 word families), and no word in the text was beyond the 5,000 word families (see figure below).

Freq. Level	Families (%)	Types (%)	Tokens (%)	Cumul. token (%)
K-1 :	89 (71.2)	100 (72.99)	199 (81.9)	81.9
K-2 :	23 (18.4)	23 (16.79)	30 (12.3)	94.2
K-3 :	9 (7.2)	9 (6.57)	9 (3.7)	97.9
		Coverage 95		
K-4 :	3 (2.4)	3 (2.19)	3 (1.2)	99.1
		Coverage 98		
K-5 :	1 (0.8)	1 (0.73)	2 (0.8)	99.9

Text 4: 98% lexical coverage was achieved with 4,000 word families, and only two words were beyond this level (*cognition* and *neuroscience*) (see figure below).

Freq. Level	Families (%)	Types (%)	Tokens (%)	Cumul. token (%)
K-1 :	70 (69.3)	77 (66.96)	188 (77.0)	77.0
K-2 :	19 (18.8)	21 (18.26)	32 (13.1)	90.1
K-3 :	9 (8.9)	12 (10.43)	13 (5.3)	95.4
		Coverage 95		
K-4 :	1 (1.0)	1 (0.87)	7 (2.9)	98.3
		Coverage 98		
K-5 :				
K-6 :				
K-7 :				
K-8 :	1 (1.0)	1 (0.87)	2 (0.8)	99.1
K-9 :				
K-10 :				
K-11 :				
K-12 :				
K-13 :	1 (1.0)	1 (0.87)	1 (0.4)	99.5

Thus, the above claim made by the authors should be reviewed, since your study cannot inform about whether 8,000-9,000 word families are needed to understand the majority of an authentic text. Rather, your study shows that, for these texts, learners required only a vocabulary size of between 3-4,000 word families in order to achieve the recommended lexical coverage for successful text comprehension (i.e., 98%). Thus, the results of this study seem to be in line with this claim that 95-98% lexical coverage of a text (whatever vocabulary size that represents in the contrived texts) is needed for adequate comprehension and inferencing to occur.

Thank you very much for these detailed comments and the lexical coverage analysis. We were not aware of the vocabulary profiler in Lextutor and believe that the addition of the lexical coverage analysis clearly benefits the paper. Out of curiosity and to understand how the profiler works, we also ran the vocabulary profiler on our four texts (including the title and using all the default options). Since this yielded slightly different numbers (though the same overall results in terms of the number of word-families needed for 95-98% lexical coverage), we are including our results here:

Text 1: *3,000 word families needed for 95% text coverage.*

Freq. Level	Families (%)	Types (%)	Tokens (%)	Cumul. token (%)
K-1 :	93 (74.4)	106 (72.11)	200 (80.6)	80.6
K-2 :	22 (17.6)	24 (16.33)	29 (11.7)	92.3
K-3 :	7 (5.6)	7 (4.76)	7 (2.8)	95.1
Coverage 95				
K-4 :	2 (1.6)	3 (2.04)	3 (1.2)	96.3
K-5 :	1 (0.8)	1 (0.68)	1 (0.4)	96.7

Text 2: *3,000 word families needed for 95% text coverage and 4,000 word families for 98% text coverage.*

Freq. Level	Families (%)	Types (%)	Tokens (%)	Cumul. token (%)
K-1 :	76 (63.3)	88 (65.19)	180 (77.6)	77.6
K-2 :	27 (22.5)	29 (21.48)	35 (15.1)	92.7
K-3 :	10 (8.3)	10 (7.41)	10 (4.3)	97.0
Coverage 95				
K-4 :	4 (3.3)	4 (2.96)	4 (1.7)	98.7
Coverage 98				
K-5 :	3 (2.5)	3 (2.22)	3 (1.3)	100.0

Text 3: *3,000 word families needed for 95% text coverage and 4,000 word families for 98% text coverage.*

Freq. Level	Families (%)	Types (%)	Tokens (%)	Cumul. token (%)
K-1 :	88 (71.0)	99 (72.79)	181 (80.4)	80.4
K-2 :	23 (18.5)	23 (16.91)	30 (13.3)	93.7
K-3 :	9 (7.3)	9 (6.62)	9 (4.0)	97.7
Coverage 95				
K-4 :	3 (2.4)	3 (2.21)	3 (1.3)	99.0
Coverage 98				
K-5 :	1 (0.8)	1 (0.74)	2 (0.9)	99.9

Text 4: *3,000 word families needed for 95% text coverage and 4,000 word families for 98% text coverage.*

Freq. Level	Families (%)	Types (%)	Tokens (%)	Cumul. token (%)
K-1 :	69 (69.0)	76 (66.67)	170 (75.2)	75.2
K-2 :	19 (19.0)	21 (18.42)	32 (14.2)	89.4
K-3 :	9 (9.0)	12 (10.53)	13 (5.8)	95.2
Coverage 95				
K-4 :	1 (1.0)	1 (0.88)	7 (3.1)	98.3
Coverage 98				
K-5 :				
K-6 :				
K-7 :				
K-8 :	1 (1.0)	1 (0.88)	2 (0.9)	99.2
K-9 :				
K-10 :				
K-11 :				
K-12 :				
K-13 :	1 (1.0)	1 (0.88)	1 (0.4)	99.6

Overall, we now state in the methods section of the paper that 3,000 word families were needed for 95% lexical coverage and 4,000 word families for 98% coverage and that only two words were beyond the 5,000 word family level. As requested, we have also changed our claims in the discussion based on the results from the lexical coverage analysis. Specifically, we now provide rough range estimates for how word family knowledge may translate into vocabulary size measured in lemmas (as in the current study). Based on these estimates, we suggest that our average participant likely had about 95% of text coverage for the particular texts chosen, which according to on Hu and Nation (2000) leads to adequate comprehension only in a minority of cases and according to Laufer and Ravenhorst-Kalovski (2010) corresponds to "minimal comprehension". That is, we could not confirm the reviewer's comment that Nation (2006) "claims that 95% lexical coverage is enough for adequate comprehension of the text (~5,000-6,000 word families for authentic texts on a variety of topics)". We have therefore substantially weakened our original claim, but not fundamentally changed it, and we hope that our current discussion is sufficiently nuanced and convincing.

Finally, regarding the results, the authors rightly compare the known words in the pre-test and the delayed post-test, and subtract the former from the latter to report relative gains. However, there is no report of the words known and unknown by the participants during the training sessions. For the sake of transparency, it would be interesting to report how many of the target words the learners reported as "known" during the training session, and how this compares with the learning retained 2 weeks later. This would allow the readers to better understand whether the learners retained the same amount of knowledge they reported during the training or indeed this knowledge was significantly higher in the delayed post-test, which would emphasise the effectiveness of the VLSs.

Thank you for pointing this out. We have now added information about how many words learners reported as known during training and how many words of those they reported as known they actually knew. Furthermore, we have provided an additional analysis to gauge how words reported as known and actually known during the training session compared to words known in the pre- and delayed post-tests. As we now report, the results revealed that participants knew significantly more target words in the delayed post-test than during training. This confirms that their knowledge of target words was significantly higher in the post-test that during training. In addition, participants reported knowing significantly more

words during training than they actually knew during both pre-test and training, indicating that they over-reported their word knowledge. Overall, participants' word knowledge in the delayed post-test significantly exceeds their knowledge during both pre-test and training, even though they over-reported their knowledge during training. We have also added information about these results in the discussion section, and hope that this sufficiently emphasises the effectiveness of the VLS.

Overall, the authors have made significant changes and improvements on the reviewed manuscript. Thus, once the comments made above are addressed, the paper should be ready for publication.

We hope that we have successfully addressed the above comments and that the paper is now ready for publication.

line 110: *8000 and 9000 words* --> word families. Also, it should be made explicit in the manuscript that these numbers were proposed for adequate comprehension of authentic texts (i.e., designed for the consumption of speakers of that language, not for learners) on a variety of topics. Thus, the vocabulary size to achieve 98% coverage in semi-authentic or contrived texts is expected to be much lower. This is important to be included also in the relevant discussion section.

Thank you for pointing this out. We have corrected 8000 and 9000 words to 8000 and 9000 word families. In addition, we have clarified in the introduction that the 8000 to 9000 word families refers to comprehension of authentic texts, and further added the information that 3000 word families have been suggested for adequate comprehension of simplified texts. Finally, have made substantial changes to our discussion of text coverage, based on the number of word families needed for 95% and 98% text coverage for the particular texts used in the study and our rough estimates of how these numbers of word families relate to lemma-based vocabulary size. As the reviewer has pointed out, the relationship between vocabulary size and text coverage is not straightforward, and we have therefore tried to be sufficiently nuanced in the claims that we are making with respect to text coverage and vocabulary size.
