# Supplementary material for: Exploring the effect of lexical inferencing and dictionary consultation on undergraduate EFL students’ vocabulary acquisition
Source: PLoS One. 2020 Jul 30;15(7):e0236798. doi: 10.1371/journal.pone.0236798 (PMC7392316; doi:10.1371/journal.pone.0236798)
Supplement: Attachment — Submitted filename: ResponsesToReviewers_v2_AA_AF.docx [file pone.0236798.s003.docx]

*We would like to thank the reviewers for their valuable comments. We believe that the comments have improved the paper substantially. We also believe that we have addressed all the reviewers’ concerns and hope that the article is ready for publication. Please also note that we have added an ethics statement to the methods section, in accordance with the journal requirements.*

Both Reviewers stated that the vocabulary size increase addressed under the research questions 4 and 5 is not easily acceptable. They both pointed out that, as you yourself mentioned in the manuscript, it might be merely due to the fact the learners took the same test twice. If you choose to revise your manuscript, I strongly request that you focus on the research questions 1, 2, and 3 (and remove 4 and 5). The two reviewers also request that some methodological details (e.g., target words, texts, dictionary) should be provided. I agree.

*Thank you for this suggestion. We have removed research questions 4 and 5, and we are now providing the requested methodological details.*

Finally, there are some more minor issues:

line 52: VLS strategies
-> VLS (because S stands for strategy)

*Thank you. Fixed.*

line 222: Likert scale always
-> scale: always

*Thank you. Fixed.*

line 208: relevant for the current
-> relevant for the current study.

*Thank you. Fixed.*

lines 243, 247: "family = poisson"
-> These analyses are not for count data. Please reconsider or justify your analyses.

*These analyses are for count data. We apologize if this was not clear. We have added information that hopefully clarifies that both analyses are for count data.*

line 280: "used as a general estimate of proficiency"
-> What kind of proficiency are you talking about here? Did you use GPAs as a general estimate of language proficiency?

*We apologize for the confusion. We have now clarified that the GPA was used as a general measure of English language academic performance and we have added some relevant references.*

line 303: "at the same time"
-> This sounds slightly misleading. What about "within the same session"?

*We actually mean “at the same time”, not “within the same session”. We have therefore added some more information and hope that this is no longer misleading.*

line 348: "Participants' average ....
-> I found this line slightly awkward and difficult to understand.  You are talking about the difference in proficiency... are you?

*We are not talking about a difference in proficiency here, but rather about how participants, on average, rated their own proficiency. We have slightly reworded this and hope that it is now easier to understand.*


Reviewer #1: Review of PONE-D-19-34526

Overall I find the study well designed and executed and the paper well-presented and thorough. I find the research questions to be answered appropriately by the method and analysis, though I am a little more skeptical than the authors about the reasons for increased vocabulary size over the duration. My hunch is that it is primarily a practice effect from taking the same test twice. I feel this makes RQs 4 and 5 somewhat unnecessary. Nevertheless, the authors’ approach to these questions and the discussion that they provide is interesting and well supported. Therefore, I recommend minor revisions but would suggest some more hedging and consideration of the counter-arguments regarding the increase in vocabulary size.

*Thank you for your kind comment that our discussion of RQs 4 and 5 was interesting and well supported. Nevertheless, and as suggested by the editor and reviewer 2, we have removed RQs 4 and 5 from the paper.*

Major comments
My major concern reading the paper was the increase in vocabulary size (addressed in RQ4 and RQ5). The average increase of 506 words over five weeks is barely believable in terms of normal second language word learning, but when considering that this increase is actually derived from an increase in the average score of just five words (5.06 words, without considering distractors), it becomes more realistic. The authors provide a very well-researched and argued discussion (line 648~) of this finding, which makes the finding reasonably palatable. Nevertheless, it requires a little more explanation. At present, only one sentence mentions “Finally, participants completed the same test twice within a period of five weeks, which may have somewhat inflated the scores of the second test” (lines 668-669). To me, this is what I assumed while reading the paper, from the RQs onwards. Basically, I was puzzled why vocabulary size was measured twice in such a short duration using exactly the same test – it seems obvious that the test itself would play some role in learning, especially if it contains the same items. The first test provides an opportunity to notice words that are only partly known or completely unknown, followed by potential opportunities to learn these words during the learning period, resulting in an overall increase in vocabulary size on the second test. This narrative does seem to fit with the research though – that is, focused training on vocabulary learning should in fact provide this kind of learning opportunity. Nevertheless, I think one sentence does not quite do justice to the most likely reason why such a large increase in vocabulary size was observed, that is, a practice effect.

*Thank you for this comment. We agree that we did not sufficiently discuss the (likely) possibility that much of the increase in vocabulary size was a learning effect. As mentioned above, we have opted to remove RQs 4 and 5 from the paper rather than provide a more in-depth discussion of a possible practice effect. We chose this route for two reasons: it was suggested by the editor, and reviewer 2 was quite a bit more critical about RQs 4 and 5.*

*Just as a piece of information: We chose to use the same test twice because we didn’t think that scores from two different tests would be comparable (one test may be harder than the other, influencing the size of the learning effect). We did briefly consider using two different tests and counterbalancing them, so that half the participants in each group take test A as a pre-test and test B as a post-test, and vice versa for the other half. However, this was not feasible within our logistic constraints for data collection.*

Also, on re-reading the Method section I noticed on line 276 that the formula may partly explain the increase in vocabulary size over the duration. Incorrect guessing (i.e., selecting pseudowords) is penalized in the formula; therefore, it is plausible that the increase in vocabulary size over the five weeks may also be partly due to reduced guessing (or increased awareness of) pseudowords the second time that participants took the test. Basically, this adds weight to the counter-argument that the increase in vocabulary size was due to a practice effect of taking the same test twice, rather than a real increase in word knowledge over the period.

*Yes, indeed, thank you for pointing this out.*

Minor points

- line 89 - change ‘brain” to ‘lexicon’

*Thank you. Fixed.*

- Could the target/control words be included as an appendix?

*Yes, of course. We have now included the target/control words in an appendix (Appendix A). We also noticed that four of the target words did appear in the reading texts, but were not underlined and thus not trained during the main training sessions. We have therefore deleted the following about the control words from the text: “and did not occur in any of the materials used for the training sessions”. Please note that, if anything, participants in the current study may have learned these four words better than the remaining 20 control words, which were neither trained nor appeared in the training materials. This, therefore, does not affect the overall results of RQ1. If anything, these four control words may have led us to underestimate the size of the learning effect that we found for control words compared to target words.*

- Were any of the items borrowed words (loanwords) sharing form and meaning in Arabic and English? I assume not, but this should be mentioned at some point when describing the materials because cognates/loanwords are often easier to learn than non-cognates/non-loanwords.

*Thanks so much for pointing this out. We have added information that none of the target or control words were cognates with or loanwords from Arabic.*

- Replace ‘mean’ with M, ‘estimate’ with β, and ‘std.error’ with SE in the text?

*We have replaced ‘mean’ with M where appropriate (i.e. we replaced instances such as ‘mean =’ with ‘M =’, but kept instances of ‘mean’ in the running text and when ‘mean’ modified a noun, as in ‘mean age’ or ‘mean word lengths’). We have also replaced all instances of ‘estimate =’ with ‘β =’ and all instances of ‘std. error’ with ‘SE’.*

- Line 231 - why was a GLM with quasipoisson distribution used instead of a non-parametric two-group test (e.g. wilcoxon test)?

*We used a GLM with a quasipoisson distribution because the data are count data (as the frequencies are counts of how often the words appear in the corpus) and the data were overdispersed. In this case, a quasipoisson rather than a poisson distribution is recommended. We have also run a Wilcoxon signed rank test, as suggested, which yields the same results as the GLM with a quasipoisson distribution (i.e. the corpus frequencies of the target and control words do not differ statistically significantly, W = 240, p = 0.33), but we continue to report the GLM with a quasipoisson distribution.*

- L243 - as above, why was a GLM with Poisson distribution used instead of t-test? I think a sentence should be provided explaining use of GLMs for these two-group comparisons.

*We have used a GLM with a Poisson distribution here as the data are count data. We have added information to the text clarifying that we are dealing with count data here. For all GLMs in this section, we have also added whether we are dealing with count data or overdispersed count data, as this affects whether we are using a poisson distribution or a quasipoisson distribution. We hope that this clarifies things. Again, a t-test yields the same results (t = -0.48, df = 45.55, p = 0.64), but as above, we would like to stick to our original analyses and hope that we have sufficiently clarified why.*

- L266-268 this sentence needs correcting for grammar

*Thank you. We have fixed this.*

- L307 - avoid contractions

*Thank you. We have fixed this.*

- L339 - ‘test session’ should be ‘training session’ I think

*Thank you. We have fixed this.*

- L363 - participants knew M=9.2 of 24 target words before training. Describing this as a ‘minority’ is true, but it is still a large proportion of target words that cannot be learned because they are already known.

*We apologize for the confusion here. We realize that we talked about these mean values in terms of the number of words known. This was not quite accurate as each correctly translated word received 2 points in the scoring system that we used. Thus, M=9.2 actually refers to approximately knowing 4.6 words (i.e. half) of the 24 target words on average. We have decided to fix this by amending the scoring system, so that correct translations now yield 1 point, partially correct ones yield 0.5 points and incorrect ones yield 0 points. Thus, the reported average values now more closely correspond to the average number of words known. We have re-run all the statistics using the new scoring system. That said, the number of known words is higher than expected based on the norming study, where participants, on average, knew fewer than three of the 24 target and 24 control words. We have therefore pointed out this discrepancy in the paper. However, we hope that the amended point distribution in the scoring system (where 1 fully known word now receives a score of 1) shows that participants did indeed know only a minority of target words (on average 4.6 out of 24).*

Reviewer #2:

This study is interesting and generally well though through, and there is certainly merit in examining the effect of these two very popular learning strategies in L2 vocabulary development. However, the manuscript in its current form does not meet the quality requirements to warrant publication in PLOS ONE. The paper attempts to address many research questions, which consequently has affected the coherence of the paper and the discussion of the main aim.

*Thank you for pointing this out. To reduce the number of research questions addressed, and as suggested by the editor, we have removed research questions 4 and 5 from the paper. We hope that this addresses the concern of too many research questions.*

More importantly, there are certain methodological issues that required more rigorous control and consideration and have affected the quality of the results and the discussion.
These and other issues are described below in more detail, along with comments and suggestions on how they might be addressed in order to warrant publication in future attempts.

*Please see our responses below. We have addressed all the methodological issues below in detail.*

Literature Review

One of the first matters that arose when reading this manuscript was the terminology employed to refer to the two learning strategies. While ‘lexical inferencing’ is sometimes used in the field interchangeably with ‘guessing from context’, I believe that using the term ‘lexical translation’ to refer to ‘dictionary consultation’ is misleading and indeed incorrect. Lexical translation involves students encountering the L1 form of a word and producing its L2 form, or vice-versa. In consulting a dictionary, learners do not have to recall any knowledge, as it is already provided to them, but simple search for the correct answer. Thus, for the sake of accuracy, the term ‘lexical translation’ should be avoided throughout the paper and instead refer to ‘dictionary use’ in future publication attempts.

*Thank you for pointing this out. We are now referring to dictionary consultation rather than lexical translation throughout the paper.*

The literature review in general requires some reorganisation and refocus in order to lead the reader more coherently to the main research question of the study. For example, since the key objective of the paper is comparing two vocabulary strategies, the taxonomy mentioned in line 46 should be discussed more thoroughly, making comparisons between other learning strategies and the two adopted in this study, and then concluding why these two and no other strategies were selected in this research.

*Thank you for this suggestion. We have generally reorganized and refocused the introduction based on the comments below. However, we disagree with the suggestion to discuss Schmitt’s (1997) taxonomy “more thoroughly,* *make comparisons between other learning strategies and the two adopted in this study, and then conclude why these two and no other strategies were selected in this research”. Schmitt (1997) lists over 50 vocabulary learning strategies, and many of them deserve further investigation. We believe that comparing the two strategies we have selected with other learning strategies (even if we do so for only a subset of the 50+ strategies) would not “lead the reader more coherently to the main research question of the study”. On the contrary: We believe that doing so would add a lot of tangential information to the paper. This is why we deliberately focused on just the aspects of Schmitt’s taxonomy that are relevant to the two VLS that we have selected. After all, our research question is not “What are the two VLS most worthy of further study and why?” To address the reviewer’s point, we have therefore instead briefly added our motivations for investigating these particular VLS: They are among those that learners have reported using most frequently in previous studies, and previous studies* *have yielded conflicting results in terms of the learning effect that both strategies yield.*

Regarding dictionary use, the literature review should discuss the differences between using monolingual or bilingual dictionaries as vocabulary learning strategies according to research. This is a key strategy in the study, and thus should be thoroughly reviewed, while focusing on the discussion of bilingual dictionaries, as it is the one used in this study. Also, for the studies reported in section starting in line 96, it would be useful for the reader if you could specify whether they were using mono or bilingual dictionaries.

*We now present research on the differences between using monolingual and bilingual dictionaries as vocabulary learning strategies, with a focus on bilingual dictionaries. In addition, we have specified whether monolingual or bilingual dictionaries were used in the studies that originally started in line 96 and now start in line 210.*

Section starting in line 135 does not link very well to the main aim of the essay, and thus I believe that it could be removed and this space dedicated to elaborate more on the key sections.

*Thank you for this suggestion. We have removed the section and are dedicating more space to the other section.*

Section starting on line 148 is very interesting and relevant to the VLS topic. However, the location breaks down the flow of ideas and diverts attention from the key research question in the study. Thus, I believe that it would lead to a more focused and coherent literature review section if this idea was discussed in the VLS section at the beginning of the literature review, and then specifying the different processing depths for each of the two target strategies in the sections under their terms (sections 2 and 3).

*Thank you for suggesting this. We have moved the processing depth section, so that we are now discussing processing depth in the VLS section at the beginning of the literature review. As suggested, we are also now specifying the different processing depths for each of the two target strategies in the sections under their terms, both based on the general literature and according to the Involvement Load Hypothesis and the Technique Feature Analysis.*

More importantly, since this is a lexical study, the discussion of processing depth must review the two key theories in the field regarding lexical engagement and processing: the Involvement Load Hypothesis (Hulstijn and Laufer 2001) and the Technique Feature Analysis (Nation and Webb 2011). Then the two target strategies should be analysed based on these two theories and the results of the study discussed in light of their different types and levels of engagement.

*Thank you for the suggestion. We are now reviewing both the Involvement Load Hypothesis and the Technique Feature Analysis in the introductory part of the paper. As mentioned in our response to the previous comment, we have also added information as to how these two approaches relate to the two VLS that are the focus of our study. In addition, we are now also discussion the results of our study with reference to these two theories.*

Finally, the references used in the study seem rather outdated (Kroll et al 1988; Carton, 1971). It is good practice to cite seminal papers, but I believe that more updated research in vocabulary learning strategies would improve the quality of the literature review and the discussion sections.

*Thank you for that comment. We have now updated the literature review and discussion sections to include more updated research in vocabulary learning strategies.*

Methodology

The general design of the study is adequate and well thought through, and the analyses performed are appropriate to answer the research questions. Yet, one of the issues that have affected the quality of the study is its general focus. It attempts to cover too many research questions, some of which are not theoretically or practically justified. In particular, RQs 4 and 5 seemed to me slightly off track. I think the potential future study attempt would be significantly improved if it concentrated on the first 3 RQs.

*Thank you for pointing this out. As suggested, we have removed RQs 4 and 5 from the paper, and we believe this has improved the focus of the paper.*

Another important methodological matter concerns the instruments and materials selected. First of all, the vocabulary size test chosen for this study is the XK_Lex. While I understand why this test might have been appropriate in that it is culturally targeted to your participants, checklists do not require learners to demonstrate knowledge. This means that the results are subject to a great extent to the participants’ understanding of “knowing” a word. Thus, the estimate of size provided is less reliable than if you had used a more standardised and reliable test, such as the VLT (Schmitt et al., 2001) or VST (Nation and Berglar, 2007).

*We believe that the various different vocabulary size tests currently available all have their advantages and disadvantages. We also believe that we have selected a test that fits the purposes of our study very well. As the reviewer notes, the test is culturally appropriate for our target learners. We also chose the XK_Lex over tests such as the VLT or VST for several others reasons, and we have therefore added additional information to the paper to explain in more detail why we chose this particular test. As we now explain in the “pre-test” section of the paper (please see citations there), the XK_Lex is considered to be reliable and valid and is used in various previous studies. It only takes about 5 to 10 minutes to administer (compared to the 40 to 60 of the VLT or VST), while covering all frequency bands from 1,000 to 10,000. It also uses the lemma rather than the word family (as do the VLT and VST) as the unit of word count and is less likely to overestimate vocabulary size than the VLT or VST. Specifically, Kremmel (2016) suggests that “using word families as the counting unit in vocabulary tests no longer seems viable” (p. 978) and proposes the lemma as a “reasonable balance between clustering words together to some degree while at the same time providing a higher degree of confidence that the word on the test does truly represent knowledge of the other words in that cluster” (p. 978). We therefore disagree that the XK_Lex presents a methodological concern, and hope that we have clarified why we selected it. We would also like to note that we disagree with the claim that the XK_Lex does not “require learners to demonstrate knowledge”.*

You have carefully controlled for the 48 target words in this study (24 for treatment and 24 for control) in terms of frequency, word length, PoS and derivational complexity. However, the list of target words should be included in a table or appendix for the reader to better interpret their characteristics and the results.

*Thank you for pointing this out. We have provided an appendix with the target and control words.*

Regarding the texts, very little information is provided about them, despite their immense importance and effect in successful lexical inferencing.

*Thank you for pointing this out. To provide the requested information, we have added an appendix with the texts used in the study (see Appendix B).*

In the literature review you mention the need for large lexical coverage to be able to understand a text and infer unknown words successfully (e.g., Nation 2006). However, there is no mention in the methodology about how the authors ensured that the texts selected for the treatment met these lexical coverage requirements in relation to the participants’ vocabulary knowledge. If this was something the authors controlled for, it should be reported in detail giving its potential effect on the lexical inferencing condition. If not controlled for, the study should be redesigned and the data collected again ensuring appropriate lexical coverage in the target texts based on the participants’ vocabulary size.

*We agree that sufficient lexical coverage is important for text understanding and successful lexical inferencing. However, we have taken a slightly different approach of controlling for lexical coverage, and we believe that our approach is more appropriate than what the reviewer suggests. We believe that it is neither feasible nor desirable to select texts based on participants’ vocabulary knowledge. Specifically, within a given classroom, learners will differ substantially in their vocabulary knowledge. In our study, vocabulary size ranges from 1200 words to 7600 words. To ensure appropriate lexical coverage for all participants (with texts at the same time containing enough initially unknown target words), we would have needed to individually select texts for each participant based on their vocabulary knowledge, or at least for each group of participants within a certain vocabulary size range. This is not feasible, and not desirable either, as it introduces additional variability and difficulties in interpreting any gains in the knowledge of target words. Instead we chose texts that were appropriate for this level overall, i.e. for undergraduate students majoring in English. We have added this information to the paper. Instead of what the reviewer suggests, we have furthermore addressed the issue of lexical coverage directly in the statistical analyses for RQ3. As we have written in the paper, we found that the higher participants’ pre-test vocabulary size, the better they learned the words in the guessing condition. In other words, participants with higher lexical coverage learned more target words through guessing than participants with lower lexical coverage.* *To further emphasize the relationship between lexical coverage and guessing, we have now added an additional analysis to the paper that shows that participants with higher vocabulary knowledge were significantly more successful at guessing from context than participants with lower vocabulary knowledge. Thus, our results confirm previous claims that sufficient vocabulary knowledge and thus text coverage is needed for successful guessing from context.*

*We believe our approach is more in line with the research questions that we attempted to answer. Specifically, as mentioned in the “current study” section, we were interested in vocabulary learning in a situation that learners may actually encounter in the classroom, and with texts that they may actually encounter in the classroom. And in this more “authentic” situation, we find that both dictionary use and lexical inferencing perform similarly for our participant group. The method proposed by the reviewer instead focuses on how successfully participants can engage in guessing from context in an ideal situation, i.e. while reading texts that are geared towards their own personal vocabulary knowledge in terms of lexical coverage, which was not our research question. We therefore argue that there is no need to redesign the study and collect data again, as our study directly addresses lexical coverage in the statistical analysis and as the suggested changes would answer a different research question.*

Similarly, in line 69 the authors start discussing the types of cues that affect lexical inferencing. Yet, it was not reported how these cues were controlled for around the target words and the control words. The 4 texts employed should be provided as appendices for the reader to evaluate and to understand which target words appeared in each text and which lexical and contextual cues each had. Moreover, the dictionary employed by learners to complete the treatment task should be cited.

*Thank you for pointing this out. We have not controlled for cues for the target words because, as above, we believe that this is not useful considering the research questions that we wanted to answer. Specifically, the cues we mention in the paragraph and that the reviewer is referring to are intralingual cues (knowledge of the target language, and related to text coverage), interlingual cues (knowledge from the native or other languages), and contextual cues (involving world knowledge). Again, our question is not what happens when we gear the text to individual participants’ knowledge of the target language, knowledge of their native and other languages and their world knowledge. Such highly individualized instruction, if it does exist, is probably extremely rare, and we are not interested in potential vocabulary learning in ideal situations, but in vocabulary learning in a realistic learning situation, i.e. a situation that learners may actually encounter in the classroom. Please also note that cues cannot be controlled for when it comes to the control words because they appeared in isolation during the pre- and delayed post-tests. Please further note that we have counterbalanced the texts (and thus the target words and the cues associated with them) across the lexical inferencing and dictionary look-up conditions.*

*We do, however, agree that providing the texts in an appendix allows the reader to evaluate and understand which target words appeared in each text and which lexical and contextual cues each had. Therefore, we now present the four texts in an appendix (see Appendix B). We also provide the additional information that learners employed a bilingual dictionary of their choice.*

As for the word translation task, the authors do not explain what kind of translation it required. It is assumed that it was L2 to L1, but this should have been explicitly mentioned. Moreover, According to vocabulary research, the different direction that a translation task can take (L2 🡪 L1 or L1 🡪 L2) affect the difficulty it poses for learners. In particular, the translation direction employed in this study (L2 form given and requesting L1 meaning to be provided) is considered easier for learners (e.g. Laufer and Goldstein, 2004), what can have influenced the results. Thus, in future publication attempts, the findings should be discussed in light of this idea.

*Thank you for pointing this out. We now mention that the translation was indeed from L2 to L1, and we mention that this is considered to be easier than translation from L1 to L2, citing the suggested literature. However, it is not clear to us how this may have influenced the results other than the task overall being easier than if participants had been asked to engage in translation from the L1 to the L2. We now additionally point out that both the lexical inferencing and dictionary consultation tasks involve translation from the L2 to the L1, so as to clarify that the direction of translation would have the same effects (being easier) on both tasks. Again, we would like to point out that this is the typical translation direction that learners encounter in the English classroom, as learners tend to engage with target language texts and translate unknown words into their native language.*

Regarding the design, the study does not include a delayed post-test session at least one week after the treatment. This limits the extent to which the proposed vocabulary gains in this study can be considered durable. I would recommend that the authors add a delayed post-test in future attempts to re-conduct this study, which could inform about the actual longer-term gains from these short sessions as well as potential differences between the two learning strategies regarding retention (i.e., the immediate gains from both can be similar, but maybe the deeper engagement required for lexical inferencing leads to more retention?).

*The study *does* include a delayed post-test session that “occurred two weeks after the second main test session”. We apologize if this wasn’t clear. To avoid confusion, we are now referring to the post-test as delayed post-test throughout the paper. Thus, the study does inform about the actual longer-term gains from these short sessions as well as potential differences between the two learning strategies regarding retention. We have chosen a delayed post-test rather than an immediate post-test both for logistic reasons and because, as the reviewer points out, immediate gains from both VLS can be similar, but the arguably deeper engagement required for lexical inferencing may lead to more retention. The results from our delayed post-test suggest that this is not the case.*

Finally, there is no mention of the time given and taken by the participants to complete the inferencing and dictionary use tasks. This information is needed for the reader to evaluate the significance and extent of the learning gains.

*Thank you for pointing this out. We have added the requested information to the “Reading texts” part under “Training”.*

Results and discussion

The above methodological issues have inevitably affected the results of the study and the discussion.

*As we have argued in detail above, we believe that our methodological choices should not be considered “issues” that have affected the results of the study. Rather, it seems that the reviewer was interested in different research questions, ones that we did not aim to address in the current study. Of course, methodological choices can and do affect results in all experimental studies. We believe that the changes that we made to the paper have addressed how some of our particular choices may have affected the results, and we hope that we have sufficiently explained our choices in the responses above and in the changes to the paper.*

Firstly, while the participants have been reported as advanced learners by the authors and the learners’ self-assessed proficiency, the results of the vocabulary size test shows that they only know 3369 lemmas. Based on this, the authors might want to reconsider reporting them as advanced learners.

*Thank you for this suggestion. We are now pointing out this discrepancy in the paper (see section on the participants’ English language profile).*

More importantly, this finding raises concerns regarding the difficulty of the four texts employed in the study and the level of lexical coverage of these texts by the participants. Did the texts meet the 98% lexical coverage required for successful guessing from context? Since the texts have not been provided, it has not been possible for me as a reviewer to check this, but it is likely that the lexical inferencing findings might have been affected by the participants’ low vocabulary level. This should be checked by the authors and discussed in future publication attempts.

*This is a good point that we now address in the discussion. Specifically, we discuss the lexical coverage of the texts with regard to our participants and the range of vocabulary knowledge that they had. Additionally, we discuss how this may have affected successful guessing from context. Note, however, that our results indicate that it is not only successful guessing from context, but also learning through dictionary usage, that is affected by vocabulary size. Thus, we believe that lexical coverage is not (as we have at length argued above) a methodological problem of the study, but instead we provide data about how vocabulary size (and as the reviewer has pointed out, therefore lexical coverage) affects both learning through inferencing, but also (and we believe this is a novel result) learning through dictionary use. It seems that lower vocabulary size (and thus lower lexical coverage) leads to less vocabulary acquisition for both lexical inferencing and dictionary consultation. As already mentioned, the texts are now provided in an appendix.*

The results of learning gains from lexical inferencing and dictionary use are interesting, and their discussion would benefit from relating the findings to the Involvement Load Hypothesis, as suggested above.

*Thank you for suggesting this. We have now related the finding to the Involvement Load Hypothesis (and to the Technique Feature Analysis, as suggested above).*

Moreover, I think it would enhance the discussion if the authors reported which words were typically learnt by most participants and interpret the findings in light of the contextual and linguistic cues included in the relevant texts were they were learnt. Did those words share common cues in the text? If so, which?

*We have looked at the words that were learned more easily by participants, as well as those that were more difficult to learn. And there are two things that stick out: First, participants seemed to have particular trouble with words that appeared in a list (such as “Cognitive processes include perception, thinking, problem-solving, memory, language and attention”). Some of the words that were easiest to learn were attributive adjectives (such as “diverse issues” or “glamorous magazine”). We have added this information to the discussion section.*

Regarding the discussion of the findings from RQ2, authors state “Our findings indicate that both VLS methods lead to higher learning than mere incidental learning”. I think it is misleading to use the term ‘incidental learning’ here when referring to control words, since this study was not comparing deliberate vs. incidental learning of words. In incidental learning situations there are not any enhanced/underlined words in the texts encountered by learners, whereas in your texts the target words were underlined. Thus, even though the control words were not enhanced in any manner, this cannot be considered lack of “incidental learning” as the students’ attention was drawn to some specific words, and thus they probably did not even notice non-underlined words. Therefore, comparing the results of vocabulary learning strategies to incidental learning in this study is not accurate.

*Thank you for pointing this out. We have rephrased all mentions of incidental learning.*

Finally, the results regarding the increase of vocabulary size and factors influencing this seem rather problematic to me. It seems quite unlikely that learners’ vocabulary size increases about 500 lemmas in 5 weeks, particularly given that the participants average vocabulary size is 3331 lemmas after multiple years of English learning and University instruction in English. A more likely explanation for this result would be the test-retests effect and the fact that the test involves only a checklist and no demonstration of knowledge, which means that participants might tick extra words in the post-test because they remember seeing them in the pre-test session even if they are unsure of their meaning or use. Thus, I believe that in future publication attempts the findings from RQs 4 and 5 should be taken with caution, and the discussion in lines 651 to 670 revised.

*As requested above, we have removed RQs 4 and 5 from the paper, so the test-retest effect is no longer an issue. We also disagree that the XK_Lex “involves no demonstration of knowledge”, and have therefore provided additional information to justify the use of this particular test.*

Overall, while the main research questions and ideas covered in this study are interesting and worth investigating, the current manuscript requires significant methodological changes for it to be considered for publication in future attempts.

*We agree that the questions we have asked in this study are interesting and worth investigating. As we have mentioned above, we argue, however, that the study does not require any of the methodological changes suggested by the reviewer. We hope we have now clearly explained our methodological choices and the reasoning behind then, and we hope that the manuscript is now ready for publication.*

Minor considerations and typos.

Abstract 🡪 the abstract says that 63 advanced learners participated in the study, but line 194 states that the final pool included only 61. The abstract information should include the final number of analysed data.

*Thank you for catching this. We have corrected this.*

Line 48 🡪 add “employed to remember”

*Thank you. We have fixed this.*

Line 60 🡪 add “lexical text coverage”

*Thank you. We have fixed this.*

Lines 66, 69 and throughout the text 🡪 number of reference in brackets does not match those in the bibliography.

*Thank you for pointing this out. We have gone through the entire paper and made sure that references in brackets match those in the bibliography throughout.*

Line 85 🡪 add “bilingual dictionary usage”

*Thank you. We have fixed this.*

Line 208 🡪 add “current study”

*Thank you. We have fixed this.*

Line 211 🡪 since you state later on (line 341) that the results of these VLS questionnaire are beyond the scope of this study I wonder whether you should mention it here at all. It can cause confusion to the reader and doesn’t add anything to this study.

*We would like to keep this information. It is very brief, and we would not want the methods section to potentially suffer from incomplete reporting. Thus, for transparency reasons, we have not made any changes here.*

Line 257 🡪 insure 🡪 ensure

*Thank you. We have fixed this.*

Line 428 🡪 knew during training 🡪 before training

*Thank you. We have fixed this.*

Line 476 and 478 🡪 vocabulary size, not ‘overall vocabulary knowledge’

*Thank you. We have fixed this.*

Lines 728-733 🡪 there is repetition of the same study (Tanyer and Ozturk)

*This part of the paper has been deleted, so this is no longer an issue.*
